# Community influences on adolescents’ use of home-brewed alcohol in rural South Africa

**DOI:** 10.1186/1471-2458-12-642

**Published:** 2012-08-11

**Authors:** Hans Onya, Abebe Tessera, Bronwyn Myers, Alan Flisher

**Affiliations:** 1 Public Health Practice and Health Promotion, School of Health Sciences, University of Limpopo, Private Bag X1106, Sovenga 0727, South Africa; 2 Department of Statistics and Operations Research, Faculty of Sciences and Agriculture, University of Limpopo, Private Bag X1106, Sovenga 0727, South Africa; 3 Alcohol and Drug Abuse Research Unit, Medical Research Council of South Africa, PO Box 19070, Tygerberg 7505, South Africa; 4 Public Health Practice and Health Promotion, School of Health Sciences, University of Limpopo, Private Bag X1106, Sovenga 0727, South Africa

**Keywords:** Community influence, Home-brewed alcohol, Adolescents, South Africa

## Abstract

**Background:**

Alcohol represents a major public health challenge in South Africa, however little is known about the correlates of alcohol use among rural adolescents. This article examines community influences on adolescents’ use of home-brewed alcohol in a rural region of South Africa.

**Method:**

A total of 1600 high school adolescents between 11 and 16 years of age participated in this study. Seven hundred and forty (46.3%) were female and 795 (49.7%) were male. Data on gender were missing for 65 students (4.0% of the sample). The age range was 11–29 years (mean age 16.4 years; Standard deviation = 2.79). A survey questionnaire on adolescent risk behavior that examined adolescents’ use of alcohol and various potential community influences on alcohol use was administered. Factor analysis was used to group community-level variables into factors. Multiple logistic regression techniques were then used to examine associations between these community factors and adolescents’ use of home-brewed alcohol.

**Results:**

The factor analysis yielded five community-level factors that accounted for almost two-thirds of the variance in home-brewed alcohol use. These factors related to subjective adult norms around substance use in the community, negative opinions about one’s neighborhood, perceived levels of adult antisocial behavior in the community, community affirmations of adolescents, and perceived levels of crime and violence in the community (derelict neighborhood). In the logistic regression model, community affirmation was negatively associated with the use of home-brew, whereas higher scores on “derelict neighborhood” and “adult antisocial behavior” were associated with greater odds of drinking home-brew.

**Conclusion:**

Findings highlight community influences on alcohol use among rural adolescents in South Africa. Feeling affirmed and valued by the broader community appears to protect adolescents against early alcohol use. In contrast, perceptions of high levels of adult anti-social behavior and crime and violence in the community are significant risks for early alcohol initiation. Implications of these findings for the prevention of alcohol use among adolescents in rural communities are discussed.

## Background

The most recent global estimates of alcohol consumption indicate that approximately 6.13 liters of pure alcohol are consumed per annum for each person aged 15 years or older, with more than a quarter of this alcohol (28.6%) comprised of home-brewed or informally produced alcohol [1,2]. This is cause for concern as conservative estimates suggest that alcohol use is responsible for 3.7% of global mortality and 4.4% of the global burden of disease [3]. When alcohol’s association with infectious disease transmission is taken into account, these morbidity and mortality rates are much higher [4]. In addition, the use of home-brewed alcohol is associated with an increased risk of harm due to the inclusion of unknown and potentially dangerous impurities and contaminants in these beverages [1].

In South Africa, a high proportion of adolescents use alcohol, with studies reporting prevalence rates for alcohol use ranging between 21.5% and 49.6% for the lifetime use of alcohol and 14.0% to 40.0% for binge-drinking [5-9]. While fewer adolescents consume alcohol than adults, there is evidence that young people in Africa who drink often do so at the same or with greater intensity than adults [2,10]. This is worrisome because of the risks associated with adolescent alcohol misuse, including risky sexual behaviors [11], tobacco smoking [12], violence and aggressive behavior, and truancy [13].

While adolescent alcohol use has been well documented in South Africa, most national or regional surveys of adolescent alcohol use have focused on urban populations. Few studies have examined patterns of alcohol use among rural populations, despite evidence that patterns of drinking differ between urban and rural adolescents. For example, urban adolescents drink mainly alcohol produced by the formal liquor industry. In contrast, rural South African adolescents drink mainly home-brewed alcohol as this is cheaper and more readily available than formally produced alcohol [13]. Despite these differences, few studies have studied factors associated with the use of home-brewed alcohol by rural South African adolescents. These factors are important to identify as they have implications for interventions to prevent or delay the initiation of alcohol use among rural youth.

Most etiological models of adolescent drinking have identified intrapersonal, interpersonal and community level risk and protective factors associated with the initiation of alcohol use. The social development model [SDM; 14] may account for how community factors influence adolescent alcohol use. According to the SDM, adolescents learn patterns of behavior, whether prosocial or antisocial, through reinforcement. However, bonding to the social group influences whether these behaviors are adopted or not. When individuals are strongly bonded to a group, they have a greater stake in conforming to the norms and values of the group [14,15]. More specifically, when adolescents are bonded to others who hold beliefs supportive of problem behaviors and who engage in these problem behaviors (such as alcohol misuse), they are likely to engage in these behaviors themselves.

Several studies conducted in developed country settings provide support for the SDM. These studies found that poor involvement with pro-social institutions; negative socialization and social bonding are risks for early alcohol use among adolescents [16-20]. For instance, a meta-analysis of thirty studies found that low levels of family support and lax controls, two dimensions of family socialization, were associated with an increased likelihood of adolescent alcohol use [17]. In contrast, alcohol-specific socialization, related to enforcing rules about not drinking, has been associated with a decreased likelihood of adolescent alcohol use [16]. A number of studies also reported that community norms and values supportive of heavy drinking and high levels of alcohol and drug use within communities promote earlier initiation into alcohol use among adolescents [18-20].

However as these studies have largely been conducted among urban youth from developed country settings, it is unclear whether these findings are relevant or can be extrapolated to a rural population from a developing country context such as South Africa. In addition, these studies have focused almost entirely on the use of formally produced alcohol and have not examined factors associated with the use of informally produced alcohol. This paper aims to redress this gap by examining community influences on the use of home-brewed alcohol among adolescents in one rural region of South Africa, namely Mankweng district in Limpopo province. More specifically, we hypothesize that adult norms supportive of alcohol use and a high prevalence of problem behavior among adults in the community will be associated with a greater likelihood of home-brew use among adolescents. In contrast, positive social bonding (as reflected through community affirmation) will reduce the likelihood of home-brewed alcohol use among adolescents. Through identifying and understanding these community influences on drinking, we hope to be able to guide the development of evidence-based alcohol prevention programmes for adolescents in rural South Africa.

## Method

### The study context

The study was conducted in the Mankweng district, which is about 30 kilometers east of Polokwane, the provincial capital city of Limpopo Province in South Africa. The province is a rural province which has a population of 5.4 million persons. Specifically, 97.1% are Black African, 2.7% are White, and 0.1% Colored (of mixed descent) and Indian/Asian respectively [21]. During the apartheid years all South Africans were classified in accordance with the Population Registration Act of 1950 into “racial groups” viz: “Black/African” (people mainly of African descent, “Colored” (people of mixed descent), “Whites” (people mainly of European descent) or “Indian” (people mainly of Indian descent). The provision of services occurred along these “racially” segregated lines [22-24]. The disproportionate provision of services to different “racial groups” led to inequalities and information is still collected along these “racial” divisions in order to redress these inequalities [25]. In no way do the authors ascribe to this classification system.

Most people in Mankweng district are Sotho, the biggest ethnic group in Limpopo Province, representing 58% of the total population, but other ethnic groups are represented in the area, with some language and cultural differences [21]. Mankweng is made up of peri-urban townships, tribal villages and informal settlements, where large families are living under more or less deprived conditions, lacking satisfactory water supply, sanitation and inadequate access to basic services [26]. Subsistence farming is common among people [27]. A significant percentage of the labor force is unemployed and people have few possibilities in general to find work. This pushes people to leave their families to seek employment elsewhere as migrant laborers [28]. This has profound implications in terms of social cohesion and many young people have to take on parental responsibilities. Thus, the population is young; with approximately 60% of the population in the province being less than 18 years old [21].

### Participants

The study population was comprised of all students attending high schools in Mankweng District in Limpopo Province, South Africa. There were 63 public high schools in this region at the time of the study. Twenty schools were randomly selected from these 63 schools, such that the probability of a school being selected was directly proportional to the number of students in the school. A total of 80 students were selected from each school; 40 were randomly selected from grade nine and grade eleven respectively. Of the students, 740 were male (46.3%) and 795 were female (49.7%). Data on gender were missing for 65 students (4.1% of the sample). The mean age for students in grade 9 was 13.85 years (SD = 1.33) and the mean age for students in grade 11 was 16 years (SD = 1.62). According to the school records, only 25 (1.5%) of the students enrolled in the selected schools absented themselves from school on the day of the research. The final study sample consisted of 1600 students. These students were equally distributed across grades 9 and 11.

### Data collection procedure

Members of the research team distributed the self-administered questionnaire (available in English and Sepedi, two of the main local languages) during regular school periods in the absence of teachers or other school personnel. The survey was administered under conditions approximating examinations. Anonymity and confidentiality were stressed. Permission for the students to take part in the study was granted by the Department of Education and Culture of Limpopo Province. The study was approved by the Ethics Committees of the University of Limpopo and the University of Cape Town. Each student who participated in the study was given a set of colored pencils to compensate for his or her time.

### Survey questionnaire

The survey questionnaire used in this study has been used with success by previous surveys of adolescent substance use in urban settings in South Africa (for example, Flisher et al., 2003) [12]. The questionnaire had three parts. Part 1 included 15 items on the demographic and socioeconomic background of the student such as age, gender, school, school grade and home language. Part 2 comprised 26 items about the use of alcohol, the use of other drugs, and psychosocial correlates for alcohol and other drug use, including community influence variables. To limit social desirability response patterns, students were asked if they had used a fictitious substance (Derbisol), and the eight students that responded positively to this item were excluded from further analyses. For this paper, we report only on the 17 community variables thought to be associated with the use of home-brewed alcohol.

These community variables included items exploring students opinions about their neighborhood such as whether students wanted to leave their neighborhood, whether they liked their neighborhood, levels of crime in their neighborhood, levels of physical violence in their neighborhood, levels of neighborhood disrepair (empty buildings), feelings of safety within the neighborhood and drug-selling within the neighborhood. For these items, possible responses ranged on a Likert-type scale from “definitely no” (1) to “definitely yes” (4). These responses were combined into dichotomous categories of “no” and “yes”.

Our community variables also examined perceived community norms around alcohol and drug use. Specifically, items examining adolescents’ perceptions of adults’ opinions regarding drug use, alcohol use, and cigarette smoking were included in the questionnaire; with possible responses ranging from “very wrong” to “not wrong at all”. Responses on these items were then combined into dichotomous categories of “wrong” or “not wrong”. We also included items thought to represent the extent to which adults engaged in antisocial behavior in the community (where antisocial behaviors were defined as involvement in illicit behaviors such as drug use and crime). These items included the number of known adult drug users, number of known adult drug dealers and number of known adults in trouble with the police. Participants were asked to estimate the number of adults known to engage in these problem behaviors. Responses on these items were reclassified into categories of “none” and “one or more adults”. Finally, we examined items thought to correspond to community affirmation of adolescents, including items such as “my neighbors notice when I do a good job”, “my neighbors encourage me to do my best”, and “my neighbors are proud of me”. For these items, possible responses ranged on a Likert-type scale from “definitely no” (1) to “definitely yes” (4). These responses were re-grouped into dichotomous categories of “no” and “yes”.

Part 3 of the questionnaire consisted of 43 items that explored the life-time and current use of home-brewed alcohol and a variety of other health-related risk behaviors. For this paper, the dependent variable was lifetime use of home-brewed alcohol, a variable with “no” or “yes” response categories.

### Data analysis

The Statistical Package for the Social Sciences (SPSS) version 17 [29] was used to conduct the analyses. First, we examined the frequency distributions of the variables of interest. As there were 17 community-level variables in the original data set, we conducted an exploratory factor analysis to try and group these variables into coherent structures. More specifically, a principal component analysis with varimax rotation was used to develop over-arching community constructs. Five factors with Eigen values greater than one were extracted from the 17 variables. These five factors were then entered as explanatory variables in a logistic regression of lifetime use of home-brewed alcohol.

## Results

In general, adolescents had positive perceptions of their neighborhoods with a large proportion (77%) not wanting to leave their neighborhood and only 19% hating their neighborhood. Only 30% of participants did not feel safe in their neighborhood (Table 1). In addition, most participants felt affirmed by their community, with 75%, 78% and 72% of students reporting that their neighbors notice when they do a good job, encourage them to do their best and are proud of them respectively (Table 1). However, students reported high levels of antisocial adult behavior in the community, with 46% and 42% of students knowing adults in the community who used drugs and dealt drugs respectively and 39% knowing adults in trouble with the police (Table 1).

**Table 1:** Descriptive statistics of community influence variables

**Variable domain**	**Variable**	**Valid N**	**% responding negatively**
**Opinion of neighborhood**	I would like to get out of my neighborhood	1547	77
	I like my neighborhood	1568	19
	Crime in neighborhood	1458	86
	Fight in neighborhood	1456	80
	Empty buildings in neighborhood	1459	83
	Graffiti in neighborhood	1466	87
	I feel safe in my neighborhood	1505	30
	Drug selling in neighborhood	1486	91
**Subjective adult norm**	Opinion of adults regarding drug use	1556	83
	Opinion of adults regarding alcohol use	1519	84
	Opinion of adults regarding cigarette smoking	1476	83
**Antisocial adult behavior**	Number of known adult drug users	1508	54
	Number of known adult drug dealers	1486	58
	Number of known adults in trouble with the police	1458	61
**Community affirmation**	My neighbors notice when I do a good job	1575	25
	My neighbors encourage me to do my best	1585	22
	My neighbors are proud of me	1585	28

### Factor analysis of community influence variables

Results of the factor analysis conducted to identify the underlying structure of the various community influence variables are presented in Table 2. Five factors, with Eigen values greater than 1, were extracted and these components accounted for 64% of the total variance in the original variables.

**Table 2:** Rotated factor loadings for measures of community influence of home brewed alcohol use

**Context**	**Factors**				
	**1**	**2**	**3**	**4**	**5**
I would like to get out of my neighborhood	.057	**.595**	.053	-.048	.151
I like my neighborhood	-.058	**-.737**	-.078	.237	.049
Crime in neighborhood	.051	**.688**	.039	-.020	**.382**
Fight in neighborhood	.063	**.641**	.110	-.069	**.381**
Empty buildings in neighborhood	.060	.020	.041	-.106	**.789**
Graffiti in neighborhood	.032	.161	.058	-.043	**.760**
I feel safe in my neighborhood	.023	**-.646**	.048	.243	.093
Drug selling in neighborhood	.037	.288	.020	-.044	**.357**
Opinion of adults regarding drug use	**.855**	.083	-.011	-.044	.067
Opinion of adults regarding alcohol use	**.923**	.046	.022	-.080	.046
Opinion of adults regarding cigarette use	**.902**	.032	.051	-.064	.035
Number of known adult drug users	.060	.027	**.829**	-.010	.074
Number of known adult drug dealers	.029	.026	**.896**	-.024	.036
Number of known adults in trouble with the police	-.032	.106	**.847**	-.007	.027
My neighbors notice when I do a good job	-.024	-.229	.040	**.736**	-.039
My neighbors encourage me to do my best	-.103	-.124	-.031	**.853**	-.064
My neighbors are proud of me	-.061	-.119	-.054	**.833**	-.119

The first factor is highly correlated with the following variables: adults’ opinion of drug use, adults’ opinions of alcohol use and adult opinions of cigarette smoking. As such it appears to represent subjective adult norms towards substance use (with higher scores representing subjective adult norms more in favor of substance use). The second factor is positively correlated with variables reflecting poor opinions of the neighborhood (e.g. high levels of crime and fighting in neighborhood) and negatively correlated with variables reflecting good opinions of the neighborhood (e.g. I like my neighborhood). This factor represents negative opinion about one’s neighborhood, with higher scores on this factor representing more negative opinions of one’s neighborhood. The third factor is highly correlated with variables relating to the known number of adult drug users, drug dealers and adults in trouble with police within the community. As such the factor appears to represent levels of antisocial behavior among adults in the community, with higher scores representing more antisocial behavior. The fourth factor is strongly correlated with neighbors noticing when a student does a good job, neighbors encouraging students to do their best, and neighbors being proud of students. As such this factor represents the degree to which the community affirms adolescents, with higher scores reflecting more affirmation. Finally, variables that loaded on the fifth factor included items referring to levels of crime and violence in the neighborhood as well as variables referring to the derelict nature of the neighborhood. Thus, this factor may represent perceptions of neighborhood disarray, with higher scores reflecting more perceived neighborhood dilapidation.

### Community influences on home-brew alcohol use

Table 3 presents results of a multiple logistic regression of the five community influence factors on the lifetime use of home-brewed alcohol. Antisocial adult behavior (Factor 3), Community affirmation (Factor 4) and derelict neighborhood (Factor 5) were significantly associated with home-brewed alcohol use. More specifically, higher scores on Factor 3 (knowing adults who engaged in antisocial behavior) were associated with a greater likelihood of adolescents’ drinking home-brewed alcohol. Similarly, higher scores on Factor 5 (perceiving one’s neighborhood as derelict) were associated with an increased likelihood of adolescent home-brewed alcohol use. In contrast, higher scores on Factor 4 (levels of community affirmation) were associated with reduced odds of adolescent home-brewed alcohol use. The predictive strength of these factors is however, relatively weak, suggesting that other variables account for much of the variance in adolescent’s use of home-brew. Despite this, the Hosmer and Lemeshow Test was significant (*χ*² = 2.74, *р* < 0.05), showing that the model is a good fit for the data.

**Table 3:** Logistic regression output

**Factor**	**B**	**S.E.**	**Wald**	**Df**	**Sig.**	**Exp(B)**	**95% C.I. for EXP(B)**							
							**Lower**	**Upper**						
FACTOR1	.012	.078	.024	1	.877	1.012	.868	1.180
FACTOR2	-.013	.076	.028	1	.868	.987	.850	1.147
FACTOR3	.336	.072	22.026	1	.000	1.399	1.216	1.610
FACTOR4	-.170	.076	5.068	1	.024	.844	.727	.978
FACTOR5	.148	.075	3.942	1	.047	1.160	1.002	1.342
Constant	−1.402	.080	310.176	1	.000	.246		

FACTOR 1 = Opinion of neighborhood, FACTOR 2 = Subjective adult norm, FACTOR3 = Antisocial adult behavior.

FACTOR 4 = Community affirmation, FACTOR 5 = Derelict neighborhood.

## Discussion

While there have been several other studies examining risk and protective factors for adolescent drinking (both in South Africa and elsewhere), to our knowledge this study is the first of its kind to examine community influences on adolescent drinking within a rural setting. We identified three community influence factors significantly associated with adolescents’ use of home-brewed alcohol: degree of antisocial adult behavior within the community, adolescents’ perceptions that their neighborhood is run-down and unsafe, and community affirmation of adolescents. These findings show that the “community” is an important influence on adolescent health behaviors in rural African communities. This is not altogether surprising given that traditionally, the health and social behaviors of individuals within rural African communities are strongly influenced by the norms and values of the broader community [30].

More specifically, we found that antisocial behavior among adults in the community significantly influenced the use of home-brewed alcohol among high school students in Mankweng, South Africa; with the likelihood of adolescents’ drinking home-brewed alcohol increasing as the number of adults known to engage in antisocial behaviors (such as drug use) increased. This finding is in keeping with findings from previous studies conducted among adolescents in urban settings in other parts of the world [31,32]. The social development model [14,15] may provide a partial explanation for this finding as this model argues that young people emulate the normative social behaviors of their primary socializing unit (including drinking and drug use behaviors). As more than 40% of young people in this study knew adults who used and/or dealt drugs in their community, a strong argument can be made for substance use being normative among adults in Mankweng. Given that the primary socializing unit in rural African contexts (such as Mankweng) is the broader community [30] and given that community norms in Mankweng appear to favor or at least tolerate anti-social behavior among community members, it is not surprising that high numbers of adolescents in this context report under-age drinking of home-brewed alcohol. This suggests that for community-based interventions to prevent and or delay the onset of drinking among adolescents to be effective, efforts also must be made to reduce substance use and other anti-social behaviors among adults.

We also found that adolescents’ perceptions about the state of their neighborhood influenced the likelihood of under-age use of home-brewed alcohol. Specifically, as adolescents’ perceptions of their neighborhood being in a derelict state and being unsafe increased, they were more likely to use home-brewed alcohol. Therefore it seems that the characteristics of the neighborhoods in which young people live play some part in influencing their drinking behavior. This is in keeping with studies conducted in other countries among adolescents which clearly point to an association between perceived neighborhood environment and substance use [33]. A partial explanation for this finding may lie in the fact that adolescents from derelict neighborhoods may feel trapped in their environments and may hold little hope for a better future. Previous South African studies have found that structural deprivation contributes to a loss of hope and a limited future orientation that is predictive of adolescent substance use [34]. Regardless of the reasons, this finding clearly points to the need for alcohol prevention strategies to tackle not only intrapersonal risk factors for underage alcohol use, but also structural risk factors (such as unemployment, crime and lack of housing) which may mediate the influence of intrapersonal risk factors on adolescents’ use of alcohol.

In addition, we found that perceived community affirmation significantly influenced the use of home-brewed alcohol among rural students; with greater perceived affirmation associated with a decreased likelihood of adolescent drinking. Communities that notice when “young people do a good job”, encourage “them to do their best” and are “proud of them” seem to protect adolescents against the early initiation of alcohol use. This may be particularly true in rural African communities, where the community plays an important role in the rearing of young people and in shaping behaviors [35]. In this context, children are viewed as belonging to the greater community rather than just the immediate nuclear family. In this socio-cultural context, feeling affirmed by the broader community potentially has a stronger influence on prosocial behaviors (such as not drinking) than in more urban and Westernized communities where the nuclear family plays more of an influence on individual behaviors [36]. Efforts to enhance feelings of affirmation may serve as an important protective device against other negative influences on adolescent behavior. Given these findings, prevention workers should consider working with existing community and cultural organizations to strengthen positive ties between adolescents and their community in a way that allows adolescents to feel affirmed and valued.

Despite these important findings, our study does have some limitations. First, we did not include out-of-school adolescents in this study, who arguably may be more at risk for substance use than in-school youth. It is entirely possible that adolescents who have dropped out of school may have an entirely different perception of adult behavior and community norms compared to adolescents who are still in school. Consequently, our findings regarding key community influences associated with adolescent drinking may not be generalisable to out-of-school youth. Second, while this study highlights some associations between community variables and alcohol use, as it is cross-sectional in nature, it is unable to examine the temporal nature of these associations. These limitations highlight the need for future research on adolescents’ use of alcohol and other drugs in rural communities. Future research should consider exploring the community-level covariates of out-of-school adolescents’ use of alcohol. In addition, longitudinal prospective studies that track adolescents and their use of alcohol over time will shed light on the temporal direction of community factors and the initiation of adolescent alcohol use. Third, this study did not include measures of alcohol availability or ease of access to home-brews within the community. It is quite possible that access to and availability of alcohol may have differed among participants which would have impacted on their use of these products. Future studies should consider including measures of alcohol availability within rural communities and ease of access to these products by young people. Finally, a qualitative study that explores how adolescents perceive their neighborhood and communities to influence their use of alcohol would deepen our understanding of this phenomenon.

## Conclusion

Despite some limitations, our findings clearly point to the influence that community-level variables have on adolescent drinking behaviors in rural communities. Our results suggest that to prevent adolescent drinking in rural communities (especially in Africa), it is essential that alcohol prevention programmes be integrated with or speak to various dimensions of the community. Such prevention programmes should include elements that work to enhance community affirmation of adolescents’ pro-social behaviors, improve adolescents’ perceptions about the state of their neighborhood (and foster a positive future orientation), and minimize antisocial behavior among adults within the community.

## Competing interests

The authors declare that they have no competing interests.

## Authors’ contributions

All authors contributed to this manuscript. HO took primary responsibility for data collection, data handling/entry, data cleaning, data analysis and the drafting of the manuscript. AT performed all complex analysis and assisted with the interpretation of results. BM was involved in result interpretation, drafting and revising the manuscript at all stages. AF helped guide the project conceptualization, planning, instrument development, and reviewed the initial draft manuscript. All authors read and approved the final manuscript.

## Pre-publication history

The pre-publication history for this paper can be accessed here:

http://www.biomedcentral.com/1471-2458/12/642/prepub
